# Evaluation of the protective efficacy of a spatial repellent to reduce malaria incidence in children in Mali compared to placebo: study protocol for a cluster-randomized double-blinded control trial (the AEGIS program)

**DOI:** 10.1186/s13063-022-06197-w

**Published:** 2022-04-05

**Authors:** Suzanne Van Hulle, Issaka Sagara, Momar Mbodji, Ghislain Ismael Nana, Mamadou Coulibaly, Alassane Dicko, Mamady Kone, Ismaila Thera, Daman Sylla, Mamadou Diango Traore, Fang Liu, John P. Grieco, Nicole L. Achee

**Affiliations:** 1 Catholic Relief Services, Baltimore, MD USA; 2 Malaria Research and Training Center (MRTC), Faculty of Medicine, Dentistry and Pharmacy at the University of Sciences, Techniques and Technologies of Bamako (USTTB), Bamako, Mali; 3 Catholic Relief Services, Bamako, Mali; 4 Department of Applied and Computational Mathematics and Statistics, University of Notre Dame, Notre Dame, IN USA; 5 Department of Biological Sciences, Eck Institute for Global Health, University of Notre Dame, Notre Dame, IN USA

**Keywords:** Spatial repellent, Malaria, Transfluthrin, Cluster randomized controlled trial, Mali

## Abstract

**Background:**

Spatial repellents have been widely used for the prevention of mosquito bites but their efficacy in reducing mosquito-borne diseases has never been evaluated in Africa. Additionally, spatial repellents have the potential of being critical tools in the prevention of mosquito-borne diseases in contexts where typical vectors control efforts such as insecticide-treated nets (ITNs) and indoor residual spray (IRS) are inaccessible or underutilized such as among displaced populations or in emergency relief settings. To address this knowledge gap, Kolondieba District, Sikasso Region, Mali was selected as a site to estimate the impact of the Mosquito Shield™, a spatial repellent that incorporates transfluthrin on a plastic sheet, on malaria-related outcomes. Over the past decade, the Region of Sikasso, Health districts of Kadiolo, Yorosso, and Kolondieba have remained among the most afflicted, characterized by an annual parasite incidence of more than 116 cases per 1000 population [1] and a *Plasmodium falciparum* prevalence rate of 29.7% [2].

**Methods:**

Cluster-randomized, placebo-controlled, double-blinded clinical trial, whereby children ≥ 6 months to < 10 years old will be enrolled and followed to determine the time to malaria infection with monthly blood samples for microscopic diagnosis. A total of 1920 subjects (HHs) will be enrolled in 60 clusters (30 spatial repellent, 30 placebo). Malaria incidence will be estimated and compared to demonstrate and quantify the protective efficacy (PE) of a spatial repellent, in reducing malaria infection. Monthly mosquito collections using CDC light traps will be conducted to determine if there are entomological correlates of spatial repellent efficacy that may be useful for the evaluation of new spatial repellents. Quarterly human landing catches (HLC) will assess the behavioral effects of the intervention.

**Discussion:**

Findings will serve as an efficacy trial of spatial repellent products for sub-Saharan Africa. Findings will be submitted to the World Health Organization Vector Control Advisory Group (WHO VCAG) for assessment of whether spatial repellents have “public health value.” Entomological outcomes will also be measured as proxies of malaria transmission to help develop guidelines for the evaluation of future spatial repellent products.

**Trial registration:**

ClinicalTrials.govNCT04795648. Registered on March 12, 2021.

**Supplementary Information:**

The online version contains supplementary material available at 10.1186/s13063-022-06197-w.

## Administrative information

Note: the numbers in curly brackets in this protocol refer to SPIRIT checklist item numbers. The order of the items has been modified to group similar items (see http://www.equator-network.org/reporting-guidelines/spirit-2013-statement-defining-standard-protocol-items-for-clinical-trials/).

**Table:** 

Title {1}	Evaluation of the protective efficacy of a spatial repellent to reduce malaria incidence in children in Mali compared to placebo: study protocol for a cluster-randomized double-blinded control trial (the AEGIS program).
Trial registration {2a and 2b}.	ClinicalTrials.gov registration Number: NCT04795648. Registered on March 12, 2021.
Protocol version {3}	February 2, 2022, Version 8.4
Funding {4}	The project under which the data reported here was gathered, “Advancing Evidence for the Global Implementation of Spatial Repellents (AEGIS),” is made possible thanks to Unitaid funding and support. Unitaid is a global health agency engaged in finding innovative solutions to prevent, diagnose, and treat diseases more quickly, effectively, and for affordable prices, in low- and middle-income countries. Unitaid’s work includes funding initiatives to address major diseases such as HIV/AIDS, malaria, and tuberculosis, as well as HIV co-infections and co-morbidities such as cervical cancer and hepatitis C, and cross-cutting areas, such as fever management. Unitaid is now applying its expertise to address challenges in advancing new therapies and diagnostics for the COVID-19 pandemic, serving as a key member of the Access to COVID Tools Accelerator. Unitaid is hosted by the World Health Organization. Additionally, SC Johnson, A Family Company (SCJ) used internal company financial resources for the development, manufacturing, delivery, and shipment of the intervention used in the study.
Author details {5a}	SVH, GIN: Catholic Relief Services, Headquarters, Baltimore, MD, USA IS, MC, AD, MK, IT, DS: Malaria Research and Training Center, Faculty of Medicine, Dentistry and Pharmacy at the University, of Sciences, Techniques and Technologies of Bamako, Bamako, Mali MM, MDT: Catholic Relief Services, Mali FL, JPG, NLA: University of Notre Dame, Notre Dame, IN, USA
Name and contact information for the trial sponsor {5b}	Dr. John P. Grieco Lead Principal Investigator, Advancing Spatial Repellents for Vector-Borne Disease Control Eck Institute for Global Health University of Notre Dame 243 Galvin Life Science Notre Dame, IN 46556 jgrieco@nd.edu 574.631.7572
Role of sponsor {5c}	As study sponsor, UND participated in study design, management, analysis, data interpretation, and manuscript development. As funder, Unitaid had no role in the design of the study and collection, analysis, and interpretation of data and in the writing of the manuscript.

## Introduction

### Background and rationale {6a}

Despite the scale-up of effective tools for the prevention and control of malaria, this disease remains one of the primary causes of morbidity and mortality in sub-Saharan Africa [3]. New tools are needed to address the threat of insecticide resistance and outdoor biting vectors and to sustain the drive to elimination. Spatial Repellents (SRs) products have shown promise as a tool to reduce biting by mosquitoes and the WHO Pesticide Evaluation Scheme has developed methods to evaluate the efficacy of new SR products [4, 5]. Despite evidence of SR efficacy in some regions like in China [6], Indonesia [7], or Peru [8], they have never been thoroughly evaluated in sub-Saharan Africa where the primary malaria vectors are strongly anthropophilic and the burden of malaria is highest. Evidence will be required to show the effectiveness of SRs across a range of malaria transmission endemicities, a range of mosquito vector species, and across different contexts of insecticide-treated net (ITN) coverage before SRs can be recommended as a tool for malaria prevention by health authorities. In addition, assessments of an SR intervention delivered under the programmatic capacity of health services have not been evaluated. It is therefore unlikely that efficacy estimates derived from tightly controlled phase III trials will be realized in program settings. This study will address the knowledge gap of whether or not SRs are effective in reducing human malaria disease in sub-Saharan Africa to inform policy makers on whether to recommend SRs as a means to further reduce malaria transmission.

Mali is resource-limited country with nearly 65% of its inhabitants living in poverty. Although tremendous gains in malaria control have been made over the years, malaria still remains a major public health concern in the country with a number of regions still registering high transmission [2]. Throughout the country, malaria remains the leading cause of morbidity and mortality, particularly among children under the age of five. Almost two and a half million clinical cases of malaria were reported in health facilities in 2015, accounting for more than a third of all outpatient visits for all age groups [9]. During the period of peak malaria transmission, the prevalence of malaria parasitemia among children aged 6-59 months was 32% based on rapid diagnostic tests (RDTs), and 36% based on microscopy [9]. Because of the high burden of malaria and the history of Catholic Relief Services (CRS) efforts in this region, the site was selected to evaluate the protective efficacy of a new SR product formulated to last up to 4 weeks for the prevention of malaria. This will be a cluster randomized control trial (cRCT), whereby children ≥6 months to < 10 years of age will be enrolled into a single cohort, randomized by cluster to receive either SR product or placebo, and followed for 24 months with intervention. While there is a risk of the SR product not providing a significant protective effect in Mali, previous studies in Peru and Indonesia using the same intervention in a 2-week formulation, and transfluthrin active ingredient (AI) have shown some promising protective effects. Based on this evidence, it is anticipated that protection in this study will benefit all household (HH) members from both genders. All blood samples will be taken for microscopic confirmation of malaria infection for the measure of time to first infection as well as the measure for overall, subsequent new infections. RDTs will be used for point-of-care diagnosis of malaria infection with microscopy used to confirm infection status. All positive malaria infections will be treated by either RDT or microscopy, clinical and asymptomatic, will be treated throughout intervention follow-up. This evaluation will serve as an efficacy trial of SR products for sub-Saharan Africa. Findings will be submitted to the WHO VCAG for assessment of whether SRs have “public health value.” Entomological outcomes will also be measured as proxies of malaria transmission to help develop guidelines for the evaluation of future SR products.

Malaria is the primary cause of morbidity and mortality in Mali, particularly among children under 5 years of age. According to the National Health Statistic (SLIS) of 2018, the Mali health facilities have registered 2,345,481 cases of malaria among them 1001 deaths [1]. Malaria places a tremendous burden on Mali’s health system as it accounts for about 37% of the motivation of the health facility consultation in Mali. Currently, the main malaria control interventions used in Mali are malaria case management, use of the ITN, IRS (although only in specific few districts of the country and not in the proposed cRCT site), Seasonal Malaria Chemoprevention (SMC) for children 3–59 months, and Intermittent Preventive Treatment in pregnant women (IPTp). Additional control measures are therefore needed to complement current interventions.

Children ≥6 months to < 10 years of age will be enrolled in a single cohort across 60 clusters (30 clusters per treatment arm, 30 clusters to receive SR intervention, and 30 clusters to receive placebo intervention). The cohort will be followed for 6 months for baseline covariate measurements and 24 months with intervention. Blood samples will be taken once every 4 weeks from all cohort subjects to test for malaria infection and whenever a subject reports a recent history of fever (within the previous 48 h). All blood samples will be taken for microscopic confirmation of malaria infection. RDTs will be used for point-of-care diagnosis of malaria infection with microscopy used to confirm infection status. All positive malaria infections, as indicated by either RDT or microscopy, clinical and asymptomatic, will be treated throughout intervention follow-up. Microscopy sample diagnosis will be used as the primary measurement for the primary (first-time infection) and secondary endpoint (overall new infections) analyses of malaria incidence. The incidence of malaria infection was measured by microscopy will be estimated and compared between treatment arms to determine the benefit of using an SR in an area with high, seasonal transmission of malaria. Entomological endpoints of exposure risk to mosquitoes will also be measured to identify entomological correlates of SR efficacy that may be useful for the evaluation of new SR products.

### Objectives {7}

#### Primary objective

The study’s primary objective is to demonstrate and quantify the protective efficacy (PE) of the Mosquito Shield™, a transfluthrin-based SR product, in reducing the incidence of first-time malaria infection in children ≥ 6 months to < 10 years of age in Mali.

#### Secondary Objectives

Secondary objectives will address key issues related to the optimization and application of SR products for public health and confirm the range of contexts within which SR PE can be achieved. Secondary objectives are:

1. Confirming and measuring the entomological correlates of reduced infection (e.g., a reduction in vector densities, mosquito infection, biting and parity rates) to set benchmark thresholds and streamline future intervention trials against other SR products by measuring only those endpoints that are correlated to PE;
2. Quantifying the efficacy in an epidemiological setting with insecticide-resistant vectors;
3. Quantifying the total number of infections averted using the spatial repellent intervention.

#### Supplementary objectives

4. To assess the relationship between the reduction in the malaria hazard rate and the decrease in mosquito CDC-LT density per night and other entomological endpoints (e.g., human landing catch (HLC), parity rate, and sporozoite positivity rate).
5. To evaluate the PE of SR for overall (total new) malaria infections, both first time and recurrent.

### Trial design {8}

The study design is a cluster randomized control double-blinded clinical trial with 30 clusters per treatment arm (SR intervention and placebo), with a 6-month baseline follow-up period, and a post cluster randomization follow-up period of 24 months with intervention. For the evaluation of the primary objective, 26 subjects (HHs) are required within each cluster factoring in a 35% loss-to-follow-up (LTFU) rate. It is expected that 80% of the baseline subjects (HHs) will continue to participate in the intervention period therefore a total of 32 subjects (HHs) will be enrolled at the start of baseline. At least one child aged from ≥ 6 months to < 10 years of age from each HH will be recruited for malaria follow-up during each baseline and intervention trial periods.

Twenty clusters (10 SR, 10 placebo) will be randomly selected to estimate the impact of the SR intervention on entomological measures of malaria transmission using CDC light traps. From these 20 clusters, a subset of 12 clusters (6 SR, 6 placebo) will be randomly selected for HLC. Light trap collections will be conducted in 10 randomly selected houses every month to assess the impact of the SR intervention on the indoor density of *Anopheles* mosquitoes. Indoor HLC will be conducted in four randomly selected houses within each of the subset 12 clusters every quarter. Four houses will be randomly selected at the start baseline, for a total of 48 sentinel houses). These 48 houses will remain fixed throughout the trial period.

## Methods: Participants, interventions, and outcomes

### Study setting {9}

**Fig. 1:**
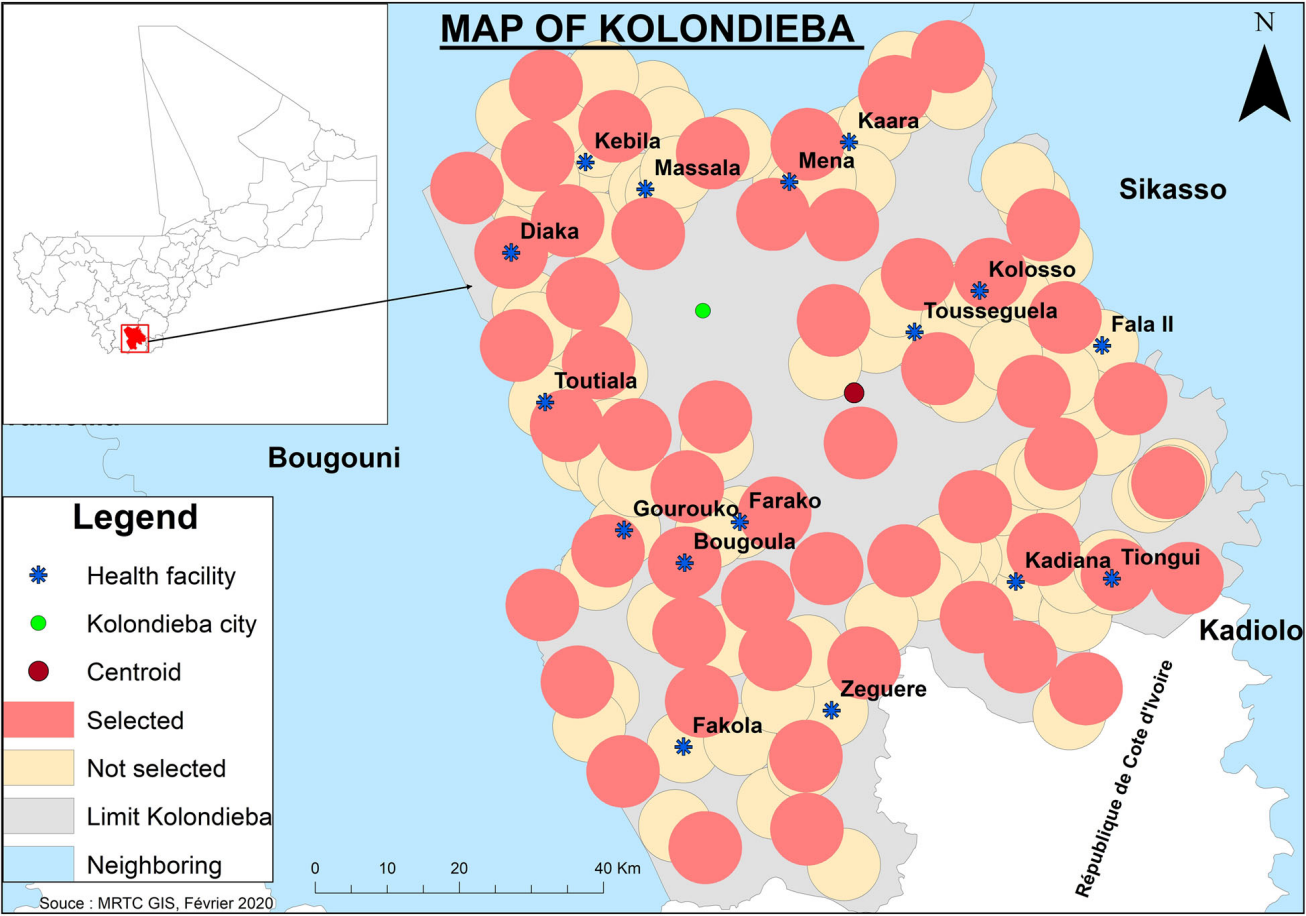
Distribution of clusters and location of health facilities in the study area

The trial will be conducted in Kolondieba District, Sikasso Region, Mali (Fig. 1). Kolondieba district has some of the highest malaria transmission rates in the country with 205/1000 population in 2018 with one malaria transmission peak period from July to November [10]. The selected district of Kolondieba has 161 villages whose GIS mapping is available through Mali's INSTAT (collected in 2009). Based on a distance of 5 km between villages to avoid clusters overlapping each other, a delineation of 60 clusters has been selected. There are a total of 17 health facilities (16 at the rural level and 1 in the town of Kolondieba) in the study area with an average distance to village/cluster groupings of 10 km.

The investigative team will monitor ongoing malaria control interventions in the clusters during the trial period to help inform in potential biases across study clusters as well as the potential augmented benefit of SR intervention to these strategies. Note too that all health interventions in the study area will be under the responsibility of the District Chief Medical Officer who is involved in this research project. Thus, the research team will be aware of all the health interventions taking place in the area and will be able to harmonize data collection methods, since the same actors are involved.

Favorable conditions for vector propagation exist in terms of temperature, vegetation, and surface water distribution with members from the 3 main malaria vector species complexes present (*An. gambiae*, *An. funestus*, and *An. coluzzii*). Wide-spread resistance to pyrethroid, organochlorines, carbamates and organophosphates has been indicated, particularly, to pyrethroids in the *An. gambiae* complex [11–13]. In the general area considered for this study, resistance has been described [14]. This could be explained by the intensive use of insecticides in agriculture. The Sikasso Region has mixed ethnicities with mainly Bambara, Senoufo, and Fulani. Housing construction is made predominately of traditional mud walls with grass or iron sheet roofing. There are limited interventions with main-stay malaria vector control methods (mainly bed net use) due to various health system challenges, although seasonal malaria chemoprevention takes place during the high transmission season as part of the national strategy. Currently, there is no planned engagement from the Ministry of Health (MOH) or partners to conduct other malaria control trials in the district of Kolondieba.

### Eligibility criteria {10}

The inclusion and exclusion criteria are listed in Table 1 below. Children aged ≥6 months to < 10 years of age who report sleeping in selected study clusters > 90% of the nights of each month and do not have any plans to travel outside the study area will be eligible for inclusion in the study. Children who have a measured hemoglobin at baseline screening/enrollment, re-screening/re-enrollment for intervention phase of < 7 g/dL, or with known chronic disease or who have signs of clinical decompensation will also be excluded from the study. During follow-up of enrolled subjects, study clinicians will have the option to conduct a Hb test for enrolled subjects when they may present signs of anemia to see if they might need additional treatment beyond malaria ACTs (if malaria infection is indicated).

**Table 1:** Inclusion and exclusion study criteria

Inclusion criteria	Exclusion criteria
Children ≥ 6 months to < 10 years of age	Children ≤ 6 months to > 10 years of age
Children with Hb ≥ 7 g/dL and no other serious illness	Children with Hb < 7 g/dL with signs of known chronic disease or other serious illness, or Hb < 6 g/dL with signs of clinical decompensation
Sleeps in cluster > 90% of nights during any given month	Sleeps in cluster < 90% of nights during any given month
Not participating in another clinical trial investigating a vaccine, drug, medical device, or a medical procedure during the trial	Participating or planned participation in another clinical trial investigating a vaccine, drug, medical device, or a medical procedure during the trial
Provision of informed consent form (ICF) signed by the parent(s) or guardian	No provision of ICF signed by the parent(s) or guardian

Persons who are participating in another clinical trial investigating a drug, vaccine, medical device, or procedure will also be excluded from the study. The written consent of the parent or legal guardian of each child will be required for inclusion in the study.

For those participants fitting the study criteria, trained study personnel will be employed to ensure the timely distribution, application, and replacement of the SR products and to perform periodic, unannounced spot checks to confirm the SR product is properly installed.

### Who will take informed consent? {26a}

The study will be explained and the consent form read in the local language to the head of HH or spouse (for SR or placebo application in the home) or to the parent/guardian of children eligible to participate in the cohort. The study will be explained in local dialect and time allowed for questions to be answered. Once all concerns are addressed, ICFs written in French (the official language in Mali) will be signed or a thumbprint taken (either on paper or electronically).

Informed consent will take place with two study populations. The study will be conducted at the cluster scale (first population) and all HHs within the selected clusters will be eligible to receive the SR product (second population). Therefore, all heads of HHs in the selected clusters will be approached and be read an ICF. Before being asked to sign the ICF, they will be asked to confirm their understanding of the ICF, to have the SR product placed in their houses and replaced every 4 weeks. The ICF will also ask that they report to the study clinic if they experience any adverse events (AEs) or serious adverse events (SAEs) of interest. AEs and SAEs of interest will be described verbally and will be provided in written form. Secondly, a cohort of children will be enrolled to estimate the impact of the SR product on the incidence of malaria disease (SR and placebo clusters). Parents/guardians of eligible children selected to participate in the cohort will be asked to provide consent. An attempt to obtain written, informed consent from both parents of the child will be made, but consent from only one parent will be required for participation (national protocols will be observed).

Separately, parent(s) and guardian(s) of eligible children aged ≥ 6 months to < 10 years will be asked to provide consent to participate in the baseline cohort with a 6 -month duration and the follow-up cohort with a 24-month duration. Children aged between ≥ 6 months and < 10 years old in identified HHs will be approached by the study team, to explain the study and ask them to voluntarily participate in the study. If subjects consent to participate, the study team will screen eligible children. After screening children for eligibility, the eligible children will then be tested and treated for malaria free of charge during the course of the study.

Parent(s)/guardian(s) who cannot sign their names will provide a thumb print on the consent form for documentation of willingness to participate, and a witness not associated with the study will sign the consent form indicating that the ICF was read, and that participation and thumb- printing were given willingly without coercion. Time will be granted to those parent(s)/guardian(s) who would wish to make consultations with their family members before signing. It will be stressed to all parents/guardians approached that their children's entry into the study is voluntary and they may withdraw from the study at any time for any reason without any penalty. In HHs with multiple children that meet the study inclusion criteria, all eligible children will be invited to participate, but only one eligible child/HH will be enrolled. Consented subjects will be assigned a subject identity code (or ID card) on enrollment. Consented participants will be screened for inclusion/exclusion criteria.

For participants who are illiterate, the staff member will ensure a literate impartial witness (a family or community member of the parent/guardian) who is not affiliated with the study is present during the informed consent process.

Copies of informed consent documents will be made available upon request to the AEGIS Program Manager (aegis@nd.edu).

### Additional consent provisions for collection and use of participant data and biological specimens {26b}

Not applicable—participant data and biological specimens will not be used in ancillary studies.

## Interventions

### Explanation for the choice of comparators {6b}

According to the WHO VCAG’s guidelines for vector control field trial design, studies should always have a control group from which data collection occurs contemporaneously with data collection from the intervention group [15]. Our trial design includes a placebo product of matched design to the Mosquito Shield™ but with inert ingredients only. The use of a placebo is generally acceptable when a placebo is compared against an intervention in combination with standard treatment [16]. Our study design will not withhold standard-of-care for clinical management of malaria nor standard-of-care vector control interventions in the placebo arm but will be monitored and recorded throughout the trial (ITNs, Seasonal Malaria Chemoprevention (SMC) for children 3–59 months, and Intermittent Preventive Treatment in pregnant women (IPTp)). This approach is aligned with WHO VCAG guidance that the control group must receive care reflecting the standard best practice interventions. The choice to use the Mosquito Shield™ product was based on this product containing the same active ingredient and design (i.e., passive emanator) as was used in clinical trials dating back to 2013 which demonstrated impact to reduce malaria and arbovirus infections [8, 17]. Thus, preliminary public health value data exists for this “first-in-kind” prototype for the SR class.

### Intervention description {11a}

The SR product used for the study will be a new formulation of transfluthrin, Mosquito Shield™, a passive emanator that releases active ingredient over a period of up to 4 weeks. SRs, like the Mosquito Shield™, are devices containing volatile chemicals that disperse in the air under ambient conditions (no requirement of electricity or heat to volatilize); they can be placed inside or around houses. The volatile chemicals introduced into the air repel mosquitoes from entering the treated space and/or disrupt human biting and feeding habits, possibly impacting their survival and reproductive behavior [4]. SR products are envisioned to complement and enhance existing vector control methods due to the continual release of volatile AI which precludes the requirement of mosquito contact with a treated surface (i.e., ITN, IRS, ULV-sprays) providing (1) protection against daytime, early-evening biting, (2) protection in enclosed/semi-enclosed and peri-domestic spaces, (3) a range of formulation options to fit context-specific application requirements thereby facilitating health systems strengthening, and (4) increase coverage of vector control over traditional methods. In addition, SR product AIs have demonstrated increased attraction to oviposition cues that could intervene in the vector life-cycle or enhance combination interventions (i.e., push-pull) that other interventions do not reach and have demonstrated effect against insecticide-resistant vector species linked to malaria transmission [18, 19].

Transfluthrin is widely used in mosquito coils and other household pest control products worldwide. The emanator consists of a pre-treated medium with a standard amount of transfluthrin that will be present throughout the treated space continuously based on a standardized 4-week replacement schedule. Products will be positioned along interior walls (approximately 2–3 m above ground) according to manufacturer specifications of 2 units per 9 m^2^. More than one emanator may be applied in a HH depending on the size of the house. A placebo product of matched design with inert ingredients will be applied similarly. Each product (SR and placebo) will have a unique code associated with an individual cluster, which will be recorded at the time of installation and replacement. Both participants and study staff will be blinded as to whether the product contains transfluthrin or is a placebo.

#### Enrollment to receive the SR product

The study product will be explained to the head of HH, as described in the main protocol, and consent for participation will be obtained prior to participation. The head of HH may be informally engaged to inquire about their perceptions and acceptability on the implementation of the project in general (not focused on the product). No routine blood samples will be sought.

#### Application of the SR during the main trial

The SR product will be replaced by paid study personnel throughout the intervention period. Both participants and study staff will be blinded as to whether the product is active (SR) or is a placebo. Products will be replaced every 4 weeks as recommended by the manufacturer. Study teams will be employed to ensure proper storage of unused products at the site and supply management and coordination for the timely replacement of products. Study teams will also perform periodic, unannounced spot checks in a random 10% of enrolled HHs during intervention on a quarterly basis to monitor SR product compliance (installation according to manufacturer specifications) and ITN usage, if available. Each product will have a unique code, which will be recorded at the time of installation and the time of replacement.

### Criteria for discontinuing or modifying allocated interventions {11b}

If a study participant chooses to end their study participation, the study staff will respect the individual’s decision without penalty. Participants who withdraw from the study will still be eligible to receive care from MOH clinicians. If a study participant no longer meets the study’s inclusion criteria and/or is based on AE and/or SAE clinical assessment, staff may terminate subject participation at any time during the trial.

### Strategies to improve adherence to interventions {11c}

In order to promote adherence to intervention protocols, study staff will be employed to ensure the timely replacement and accurate placement of the SR products. Additionally, they will perform periodic, unannounced spot checks to confirm the SR product is properly installed. If study staff observe a product has been moved after application during a scheduled product replacement, the move will be recorded for use in household compliance assessment. If necessary, study staff will re-engage with heads of households on the importance of maintaining original product placement. Overall product coverage will be estimated based on total HHs recorded having product volume at the time of replacement according to manufacturer specifications (2 units/9 m^2^).

### Relevant concomitant care permitted or prohibited during the trial {11d}

While the standard-of-care for clinical management of malaria and vector control interventions (e.g., ITNs, IRS) will not be withheld in either the SR or placebo arm, these interventions will be monitored and recorded throughout the trial. At baseline, children enrolled into the cohorts will be provided a treatment dose of artemether-lumefantrine (AL) free of charge for malaria parasite clearance and will be provided a new ITN. In addition, subjects will be provided treatment for malaria infection throughout the follow-up period. Lastly, participants will be encouraged to continue ITN use and not instructed to avoid alternative vector control tools (e.g., coils, topicals, aerosol sprays, repellents) which will allow for an estimation of the SR effect assuming all other measures are still occurring for malaria prevention, essentially providing insight on an additive benefit above that provided by currently recommended WHO malaria preventive measures.

### Provisions for post-trial care {30}

Not applicable—the study will not provide post-trial care.

### Outcomes {12}

1. The primary outcome measure of this cRCT will be the number of first-time malaria infections (*Plasmodium falciparum* (Pf)) during the intervention period (24 months) as measured by microscopy in children aged between 6 months and 10 years.

Secondary outcome measures include:

2. Number of overall new *Plasmodium falciparum* malaria infections during the intervention period (24 months) as measured by microscopy in children aged between 6 months and 10 years;
3. Number of first-time *Plasmodium falciparum* malaria infections during the intervention period (24 months) by two age groups (≤ 6–59 months old; 5–10 years old) as measured by microscopy in children aged between 6 months and 10 years;
4. Number of overall *Plasmodium falciparum* malaria infections during the intervention period (24 months) by two age groups (≤ 6–59 months old; 5 years to 10 years old) as measured by microscopy in children aged between 6 months and 10 years;
5. Anopheline-human contact (indoor and outdoor) using human biting rate (HBR) as an indicator for all anophelines and by anopheline species as measured by human landing catch (HLC) during 12-h intervals on a quarterly basis during the intervention period (24 months);
6. Anopheline parity rate as an indicator of population age structure for all anophelines and by anopheline species as measured by mosquito ovarian dissections from a sub-sample of anophelines collected during HLC procedures during the intervention period (24 months);
7. Anopheline infectivity using sporozoite rate as an indicator for all anophelines and by anopheline species as measured by laboratory detection of sporozoites in mosquito head-preps from a sub-sample of anophelines collected during HLC and/or CDC-light trap procedures during intervention period (24 months);
8. Anopheline infectivity using entomological inoculation rate (EIR) during the intervention period (24 months) as an indicator for all anophelines and by anopheline species as measured by calculating the number of sporozoite-infected anopheline mosquitoes captured per person during intervention period from HLC and/or CDC-light trap procedures;
9. CDC-light trap indoor density for all anophelines and by anopheline species as measured by CDC-light trap collections during 12-h intervals on a monthly basis during the intervention period (24 months);
10. Insecticide resistance as measured by WHO filter paper test and CDC bottle assays during the baseline (6 months) and intervention period (24 months);
11. Adverse events (AEs) and serious adverse events (SAEs) as measured by solicited and unsolicited reports during baseline (6 months) and intervention period (24 months). Mean, minimum and maximum frequency, and percentage of AEs and SAEs across clusters among enrolled subjects will be summarized by the treatment arm.

### Participant timeline {13}

The participant timeline is shown in Table 2.

**Table 2:** Study timeline

	Study period			
	Baseline	Intervention		Close-out
**Timepoint**	*T*_(Jul 2021–Jan 2022)_	*T*_(Jan 2022)_	*T*_(Feb 2022–Jan2024)_	*T*_(Feb2024–Apr2024)_
**Baseline**				
**Informed consent**	X			
**Screening**	X			
**Follow-up**	X			
**Intervention**				
**Allocation**		X		
**Informed consent**		X		
**Screening**		X	X	
**Follow-up**			X	
**Assessments**				
**Baseline final analysis**		X		
**Final analysis**				X

### Sample size {14}

Assumptions used to calculate the sample sizes will be evaluated during the baseline phase and may lead to an adjustment in the overall sample size required for the trial’s intervention phase. Since the adjustment will only utilize the estimated baseline incidence and coefficient of variance (CV) from the baseline without any intervention or randomization information, the Type-I error rate will not be inflated.

#### Primary hypothesis on first-time malaria infection

The sample size determination on the required number of subjects (HHs) per cluster for testing the primary hypothesis on PE is based on the hazard rate comparison in the proportional hazards regression model [7, 8]. With the following specifications: 1-sided type-I error rate = 5% (because the primary hypothesis is one-sided as SR is very unlikely to increase the hazard rate of malaria infection compared to placebo), true PE = 30%, a between-cluster coefficient of variance (CV) of hazard rate = 47% (based on the historical data collected from Mali) then 788 independent first-time malaria events will need to be observed to reach 80% power in testing the primary hypothesis on PE.

With a baseline first-time malaria infection hazard rate of 1.0 per person-year (ppy), 30 clusters per treatment, 26 subjects (HHs) per cluster (factoring in a LTFU rate at 35%) in each treatment arm post randomization are expected to yield 788 independent first-time malaria events within 24 months follow-up period per cohort post randomization to yield 80% power. If, by the end of the 2-year study, 788 independent malaria events are not reached, the study may extend until 788 events are collected without inflating the type I error rate in the testing of the primary hypothesis. The same number of HHs (26) will be recruited into the baseline period. It is expected that 80% of the baseline HHs will continue to participate in the intervention period.

#### Secondary hypothesis on overall malaria infection

The same sample size calculation for the first-time infection PE applies to the hypothesis on the overall malaria infection, understanding that the 80% power goal might be an understatement with the same number HHs, CV assumption, and the true PE effect as for the first-time malaria infection above, due to the following two reasons: the baseline overall malaria infection hazard rate is likely to be higher than 0.7 ppy and there is no interim analysis on the overall malaria infection.

Since the sample sizes for PE evaluations already factor in the LTFU rate, there is no need for replacement subjects unless the LTFU is larger than assumed. If replacement subjects are to be recruited, they should not have been exposed to the intervention until the time they are considered for replacement.

### Recruitment {15}

Recruitment will be done after community entry through Community Advisory Board meetings, Community Leaders, community mobilization, and sensitization. This will only be done in the study areas—i.e., in villages (clusters) surrounding the study clinics. A document describing the general information regarding the study will be given to community members during large gatherings. This document formally will introduce the study to community members by Malaria Research and Training Center (MRTC) staff after a community leader’s acceptance. After the start of the baseline, local radio spots will be used to inform community members about the details of the study and upcoming scheduled events. These radio spots are intended to reach a large audience in Kolondieba District.

## Assignment of interventions: allocation

### Sequence generation {16a}

The unit of randomization for the intervention and placebo will be a cluster. For the baseline cohort, the recruitment of participants for enrollment was based on the random selection of HHs using census mapping of the study area. The study statistician will analyze data from the baseline follow-up period to inform on potential stratification requirements prior to randomization. Criteria for stratification will be baseline malaria incidence levels and/or adult entomological endpoints. Following stratification (as needed), clusters will be allocated to receive either active or placebo treatment using a random number generator (https://www.random.org). Following cluster treatment allocation, a simple random sample will be completed for the selection of sub-clusters assigned for entomology collection. The cluster allocation code will be made available from the intervention manufacturer to the Data Safety Monitoring Board (DSMB) for use in safety assessments. The site database manager will assign a unique identification number to each household (HIN) and the site intervention administrator will coordinate the distribution of blinded active or placebo to enrolled HHs within each cluster corresponding to the pre-labeled package code.

### Concealment mechanism {16b}

The cRCT involves the Mosquito Shield™, an SR with the AI transfluthrin. A placebo, which will be identical in size, shape, and color but without transfluthrin, will be provided to control villages/clusters to ensure blinding. SR and placebo intervention will have identical packaging and will be deployed in houses by study personnel using a blinded coding scheme. To further minimize bias, an objective primary outcome measure will be used to mask all laboratory staff to the product assignment of individual clusters.

The study biostatistician will remain blinded throughout the trial, but will conduct an unblinded analysis following database lock upon completion of all data entry and resolution of standing data queries at the end of the study.

### Implementation {16c}

Intervention distribution will occur after the completion of baseline phase analyses has been completed and underlying assumptions on study power (incidence, CV) are verified. All consented HHs will have the product placed inside their homes at the manufacturer’s recommended application rate of 2 units per 9 m^2^ floor area. Trained study staff will be responsible for the management of product implementation which will include the initial deployment of product, subsequent removal, and replacement at 4-week intervals.

## Assignment of interventions: blinding

### Who will be blinded {17a}

All participants, investigators, study staff, and the biostatistician will be blinded for allocation and the trial duration.

### Procedure for unblinding if needed {17b}

#### Non-emergency unblinding of a single participant

If, because of an AE that might be related to SR product, and non-emergency unblinding of an individual participant can be considered. Site Principal Investigator (PI) will contact UND lead PIs and DSMB Medical Monitor to discuss the case and obtain agreement that the participant should be un-blinded in a non-emergency manner. If unblinding is agreed upon, the sealed (digital password protected) intervention assignment will be with the site PI but only opened by a pre-designated person external to the study (i.e., administrator) so as to maintain PI’s blinding to cluster assignments. Documentation of the unblinding will be done and subsequent follow-up memo to the study co-PIs, medical monitors, and DSMB. This unblinding should be associated with the person not the cluster or arms to control for unblinding of the cluster.

#### Emergency unblinding

If, because of an AE or SAE that might be at least possibly related to the SR product, unblinding of the participant or multiple participants can be considered. If no information is available, then emergency unblinding should be done so as not to reveal actual intervention assignments. Emergency unblinding will be considered if there is suspected unexpected serious adverse reaction to the study protocol or serious adverse reaction to AL as judged by the study physician or study safety monitor. The first alert will be raised by the study physician within 24 h of becoming aware of the event in an expedited report on the SAE to the site PI, Sponsor PI, local ethics committees, site Clinical Officer, and DMSB. The final decision for emergency unblinding will be advised by the site PI in consultation with the site Clinical Officer and/or DMSB chair.

## Data collection and management

### Plans for assessment and collection of outcomes {18a}

#### Mapping of the study area and baseline census measurements

Prior to enrollment, structures in the study area will be mapped using GPS coordinates and assigned a unique HIN. A baseline questionnaire will be administered during mapping to measure housing structure and HH demographic characteristics. Profiles of enrolled houses that could potentially confound effect on mosquitoes will be generated. Profiles will include house construction, socioeconomic status, number of inhabitants and their age, current HH vector control method(s) including ITNs.

Villages will be considered as the unit of randomization. According to Mali’s 2009 National Population Census [20], the circle of Kolondieba is comprised of 11 rural communes with a population size of 201,456 inhabitants. This population is divided between 30,079 HHs and 14,730 family compounds, where a compound is defined as a family unit with one or more structures. A typical family compound has 5 HH members.

Clusters will be randomly allocated to intervention or placebo using standard statistical software. All HHs in each village will be invited to participate in the study by allowing study staff to place SR or placebos in their HH. SR or placebo will be placed in each HH that consents. Within each cluster, individual compounds will be enumerated and then randomly selected for inclusion in each cohort.

#### Enrollment of the cohort

A cohort of children aged ≥ 6 months to < 10 years of age will be screened and enrolled within study clusters to assess the PE effect of the SR product against a placebo control. HHs will be randomly selected from the master list of HHs obtained during baseline mapping to achieve the desired sample size.

Selected HHs based on cluster maps will be visited and if a child is present, the study will be explained and consent obtained from the child’s primary caregiver (as needed) before screening the child to verify inclusion criteria for enrollment into the study. All eligible children in a selected HH will be invited to participate but only one eligible child/HH will be randomly enrolled to participate in the study.

At screening, for baseline, and re-screening for intervention phase the child’s age and gender will be recorded and the parent/guardian (as needed) will be asked about involvement in other clinical trials, travel outside the cluster area and the ITN use, recent administration of seasonal malaria chemoprevention drugs, and anti-malarial drugs. Contact information, as available, will be captured. A fingerstick blood sample will be taken for a malaria RDT, a blood smear, and a measurement of hemoglobin.

All children successfully meeting screening inclusion criteria and enrolled into the cohort will be provided a long-lasting insecticidal net (LLIN). This will allow us to measure the added benefit of SR product above that provided by currently recommended preventive measures. In addition, at baseline, subjects enrolled into the cohort will be provided a treatment dose free of charge of artemisinin-combination treatment (ACT), artemether, and lumefantrine, according to national protocol, to clear any prepatent or patent malaria parasites. RDTs will be used for point-of-care diagnosis of malaria infection with microscopy used to confirm infection status. All positive malaria infections as indicated by either RDT or microscopy, clinical and asymptomatic, will be treated. If a subject has a RDT negative outcome but a positive microscopy diagnosis, follow-up treatment for the malaria infection will be provided to the subject within 72 h of the microscopy read.

The enrollment and pre-product introduction period (baseline) will take approximately 6 months, during which time incidence of malaria infection will be measured prior to the deployment of the study product.

#### Cohort follow-up

All efforts to overlay commencement of baseline follow-up with start of malaria transmission season will occur; however, programmatic timelines may not allow for perfect alignment. The primary goal during baseline will be to ensure capturing incidence across a range of anticipated transmission timing in order to inform on sample size assumptions prior to intervention.

At enrollment, children will be cleared of parasitemia with a treatment dose of ACT, just after deployment of the study product. This will be done so incidence of malaria parasitemia following study product deployment can be measured. The distribution of the SR will occur after the completion of the baseline period. At this time, cohort participants will be presumptively cleared of parasites with a treatment dose of AL unless they have recently been treated (within the last 2 weeks). This will be done so incidence of malaria infection following study product deployment can be measured.

During the entire study period, each subject will be asked to come to the nearest study health facility for a total of 30 scheduled clinic follow-up visits for passive malaria case detection. Scheduled clinic visits will occur one time per month during each the 6-month baseline period and 24-month period with intervention. Subjects will be visited at their home if they cannot reach the clinic for the scheduled follow-up. In addition, each subject will have scheduled HH visits conducted by study staff for active malaria case detection where blood sampling will occur only for those subjects reporting fever history. Scheduled HH visits will occur one time per month during each the 6-month baseline period and 24-month period with intervention. Lastly, subjects will be informed to make an unscheduled clinic visit any time during the trial should they experience malaria-related symptoms.

At each visit, the parent/guardian will be asked about the recent use of ITNs and other vector control interventions as well as the recent history of the child’s illness, recent travels outside the HH, and recent use of anti-malarial drugs. At every other visit (on a monthly basis), a blood sample will be taken for malaria RDT, and a blood smear for confirming malaria diagnosis. At the intervening visits, a blood sample will only be taken if the child has a recent history of fever. Total blood volume for samples at each visit will not exceed 500 μl.

During follow-up of enrolled subjects, study clinicians will have the option to conduct a Hb test for enrolled subjects when they may present signs of anemia to see if they might need additional treatment beyond malaria ACTs (if malaria infection is indicated).

RDTs will be used for point-of-care diagnosis of malaria infection with microscopy used to confirm infection status. All positive malaria infections as indicated by either RDT or microscopy, clinical and asymptomatic, will be treated. If a subject has a RDT negative outcome but a positive microscopy diagnosis, follow-up treatment for the malaria infection will be provided to the subject within 72 h of the microscopy read.

Cohort subjects who test positive for malaria by either RDT or microscopy, symptomatic or asymptomatic, during both scheduled and unscheduled visits will be treated with ACTs free of charge according to national treatment guidelines. Hospital fees will not be paid by the study unless the illness or injury is due to study product or procedures as determined by a study clinician. If there is illness or injury due to study product or procedures, fees will be paid for care at the government clinic or District or Provincial Hospital according to the clinical trial insurance policy of the trial.

#### Entomological collections

Twenty clusters (10 SR, 10 placebo) will be randomly selected to estimate the impact of the SR on entomological measures of malaria transmission. Within each cluster, light trap collections will be conducted monthly in 10 randomly selected HHs to assess the impact of SRs on the density of *Anopheles* mosquitoes indoors. The light traps will be deployed next to a person sleeping under a net and will run from approximately 6 pm to 6 am. The 10 SR and 10 placebo clusters will be randomly selected at the beginning of the study and will remain fixed throughout the study. HHs will be sampled with replacement and therefore will be eligible for repeated sampling throughout the study. HHs enrolled in the cohort study will not be eligible for mosquito sampling.

HLCs will be done indoors and outdoors in 6 intervention and 6 control clusters (the 12 clusters will remain fixed throughout the study) in four houses (randomly selected) in each cluster for the period of 2 nights (total of 48 houses across both arms) once every quarter (3 months) to determine the effect of SR on the host-seeking behavior of mosquitoes. The same clusters and the houses will be sampled each sampling period. SR and placebo clusters will be selected in pairs to reduce the degree of heterogeneity between two arms. Sentinel houses will be chosen so they are balanced as to house design and inhabitants to reduce bias due to mosquito density heterogeneity (i.e., some houses may be more attractive to mosquitoes than others). HHs with children enrolled in the cohort are not eligible for the HLCs. One pair of clusters will be sampled on any given night. Collections will start at 6 pm each night with one person inside and one person outside. The teams will collect for 45 min each hour and then take a 15-min break before starting the next hour of collection. At midnight, a new team will begin collecting and will continue until 6 am. Mosquitoes will be sorted by site, date, and time of collection. All mosquitoes will be identified to species and all female anopheline mosquitoes will be tested for the presence of sporozoites by ELISA and/or PCR. A subset of up to 30 per cluster per sampling period will be dissected for parity.

A team of 16 collectors will be hired to conduct the indoor/outdoor landing catches in 4 houses in each of 12 clusters (6 intervention and 6 control) every quarter following informed consent. Mosquito collectors will be tested for malaria if they develop symptoms and treated according to the national policy if the result is positive.

Monitoring for insecticide resistance will be done once during pre-intervention/baseline, mid- intervention (12 months) and post-intervention (within 3 months following last subject follow-up) in 4 intervention and 4 control clusters (selected by the independent statistician, blinded to investigators) spread across the study area. These data will not be used to assess the impact insecticide resistance would have on the intervention but rather to inform on the general status of insecticide resistance in the area. In each site, larvae or adults will be collected and reared in the insectary at a MRTC facility. Unfed adult females that are 2–5 days old will be tested in the WHO tube bioassay technique against permethrin to characterize resistance status. CDC bottle bioassays will be conducted, as appropriate, to assess transfluthrin susceptibility as well as permethrin resistance intensity at 1X, 2X, 5X, and 10X diagnostic dose.

Temperature and relative humidity will be recorded from inside and outdoors of HHs enrolled in entomological sampling using Hobo data logging devices. Precipitation (rainfall) data from national weather stations will also be explored for clusters within the entomology sampling frame. Aggregation of precipitation data for entomology clusters will be conducted by the treatment arm, as needed, based on limitations of geographical availability.

#### Data forms

A combination of standardized paper-based or digital forms (under Android tablets) will be used. MRTC, CRS, and University of Notre Dame (UND)/Center for Research Computing (CRC) will work together to develop the quantitative forms to be uploaded on Android phones. Entered data (entomological and epidemiological) will be automatically assessed for quality using established quality control rules, then reviewed and appended to the data already present. Data forms can be made available upon request submitted to UND. Any changes to the forms will need to be agreed by the cRCT overall CRS PI (in consultation with CRS’ technical advisors), the country level MRTC and CRS PIs, and UND’s co-PI. In the event a consensus on proposed changes cannot be achieved, UND’s PI will make the final decision.

### Plans to promote participant retention and complete follow-up {18b}

Participant retention strategies will include study staff directly visiting study participants in their HHs twice a month. Additional strategies will include participant tracing when they miss appointments, visit reminders, request participants to inform study staff of moves outside or within the study area, foster relationships with participants as they engage in ancillary care at study clinics, provision of Clinical Officer’s contact information for easy communication to alleviate any concerns, and periodic generation of retention rate to evaluate strategies. A study SOP will be developed to provide more details on retention activities to be conducted by study staff.

### Data management {19}

#### Data forms

A combination of standardized paper-based or digital forms (under Android tablets) will be used so variable codes can be cross-referenced during the final analysis. All data issuing from the electronic data collection system will follow the same data collection processes outlined in the paragraph below.

#### Data quality control and quality assurance

Data management study staff will be responsible for verifying data accuracy and assuring data collection is following standardized protocols. These activities will promote data quality and ensure the trial is performed in compliance with GCP and the applicable regulatory requirements(s). Training on data collection will occur prior to the start of baseline and throughout the trial period with refresher trainings. Standardized data collection forms will be used and source data verification will occur through three primary mechanisms: (1) Self-quality checks, making sure data forms are fully completed; (2) Data queries, quality checks on a routine basis; (3) External monitoring, by fhiClinical, the clinical research organization responsible for trial oversight. In addition, tablet-based digital forms which will be used for data entry will be custom designed to include rules and conditions for data variable responses (e.g., text responses cannot occur for a numeric value, and thresholds for numeric data).

#### Data sharing policy

Using CommCare, the study teams will collect data which will be securely stored on Android devices and synchronized to the CommCare cloud. Data will undergo an initial cleaning and de-identification by the study team, before being synced to UND’s central database. Data from MRTC to UND will be transferred through a dedicated secure FTP server with password-protected access from MRTC and CRS.

This project will generate considerable data and biological samples over the course of the 2-year study period. The data management plan will follow the guidelines and suggestions put forward by the National Institutes of Health in its online guidance document https://humansubjects.nih.gov/data_safety and by the respective institutions involved in the research (MRTC, UND, and CRS), as well as by the community of interest (comprised of colleagues, scientists working in the same field, the biomedical community researching tropical parasitic diseases, and public health officials). The goal is transparent sharing of key findings and data so that the broad impacts of the research are meaningful and useful to key stakeholders and will therefore be shared with stakeholders as may be required.

Every consideration will be given to the nature of beneficence and justice expressed in the guiding documents for research on human subjects. Biological samples of mosquitoes and parasites will be maintained appropriately to avoid deterioration. These materials will be made available to researchers upon reasonable request and with the caveat that any forthcoming publications from research on the samples should consider the original researchers and their inclusion in the resulting publications when warranted.

#### Data storage

Any data collected with paper forms (including consent forms) will be scanned and transferred to binders for storage in a secure and locked restricted access area, while all electronic captured data will be archived with a documented history of changes or corrections at the local study site. Using CommCare, MRTC (with technical support from CRS) will collect data which will be securely stored on Android devices and then synchronized to CommCare cloud at least every 14 days. MRTC will download the data from CommCare and conduct initial data cleaning and de-identification, in collaboration with CRS, before syncing it to UND’s central study database. The downloaded data will be stored on password-protected computers at the MRTC office to ensure confidentiality. Data from MRTC to UND will be transferred through a dedicated secure sFTP server with password protect access from MRTC via CommCare.

A password-protected central study database warehousing data from all sites will be developed and managed by UND and serve as a data repository and utilized for safe and confidential data storage, extraction, integration and analysis. The data warehouse and file repository will be backed up weekly at the local server level to ease recovery as needed. In addition, data is stored and backed up on CommCare cloud. Access to study data will be controlled through centralized administration and access will be granted only through the PI’s permission. Research records for all study subjects including history and physical findings, and results of consultations are to be maintained by the local site PI in a secure storage facility and by UND and CRS, for a minimum of 3 years after the end of the projects per national guidance or until notified by grantee. Data and samples can be destroyed at any given time after those 3 years.

### Confidentiality {27}

Any participant information will be confidential and will not be given to anyone who is not sensitive to the participant’s health. The results of this study will be made publicly available to sponsors of this study but personal information will not be provided to anyone.

The UND CRC will not share identifiers, but instead, use a code. The code will be kept by the UND CRC, and securely at the MRTC site according to site-specific IRB specifications and requirements for emergency situations. Raw data will be anonymized and GPS tag-blurred to remove sensitive information prior to sharing to other study sites or outside of the core study team according to local IRB requirements.

Participant privacy/confidentiality protection will be assessed during routine quality assurance activities and based on the SOPs developed for the project. A set of protocols and contingency plans for emergency paper-based and digital data destruction will be developed in order to guarantee privacy of research subjects in case of unforeseen risks.

### Plans for collection, laboratory evaluation, and storage of biological specimens for genetic or molecular analysis in this trial/future use {33}

This study does not include genetic or molecular analyses. Standardized protocols will be developed for the collection, storage, use, and post-trial destruction of blood samples collected as part of the trial.

## Statistical methods

### Statistical methods for primary and secondary outcomes {20a}

#### Primary hypothesis

H_0_: SR does not reduce the first-time malaria hazard rate compared to placebo in Mali.

H_1_: SR reduces the first-time malaria hazard rate compared to placebo (overall malaria hazard ratio between SR and placebo is <1; the expected hazard ratio is 70% or PE is 30%).

#### Secondary hypothesis

H_0_: SR does not reduce the overall malaria hazard rate compared to placebo in Mali.

H_1_: SR reduces the overall malaria hazard rate compared to placebo (overall malaria hazard ratio between SR and placebo is <1; the expected hazard ratio is 70% or PE is 30%).

#### Population for analysis

The intention to treat (ITT) analysis is the primary analysis approach for both the primary and secondary objectives. The ITT population includes the first recruited child from each recruited HH that receives at least one SR product or placebo per the cluster randomization schedule. If a recruited subject comes from a HH used for entomological data collection, that subject will be not used in the ITT analysis. The per-protocol (PP) analysis is included as a supplementary analysis for the primary and secondary objectives. The PP population includes the subjects from the ITT population that are treated following the specifications of the study protocol without major protocol deviations.

#### Statistical methods—primary endpoint (ITT population)

The baseline characteristics of the enrolled subjects, HHs, and clusters will be summarized by treatment arm. Specifically, we will examine subject age and gender at the individual level, wall type and roof type, floor height, house open eaves, # of window, # of doors at the HH levels, and cluster population and baseline overall infection incidence at the cluster level.

The primary hypothesis on PE against the first-time malaria infection will be tested by comparing the hazard rates of first-time malaria infection between SR and placebo upon the completion of the study in the ITT population via a cloglog regression for the interval-censored time to event data. The model will include the treatment arm (SR vs. placebo), relevant baseline covariates, and a random effect that accounts for the dependency among data collected from the same cluster. PE against the first-time infection is estimated by ($1-\exp\ \left(\hat{\beta}\right)\ \Big)\times$100%, where$\hat{\beta}$ is the estimated regression coefficient associated with the treatment group, with a 90% 2-sided confidence Interval (CI) based on the Wald test. The lower bound of the 90% CI corresponds to the lower bound of the 95% 1-sided CI (the hypothesis is one-sided).

#### Statistical methods—secondary endpoints

##### PE of SR against the overall (both first-time and recurrent) malaria infection

The secondary hypothesis on PE against the overall new malaria infections will be tested by comparing the hazard rates of the overall malaria infection between SR and placebo in the ITT population. using a similar approach as for the first-time infection with an additional random effect term to account for the dependency among the multiple malaria incidences collected from the same individual. PE against the overall infections is estimated by ($1-\mathit{\exp}\ \left(\hat{\beta}\right)\ \Big)\times$100%, where$\hat{\beta}$ is the estimated regression coefficient associated with the treatment group, with a 90% 2-sided CI based on the Wald test. The lower bound of the 90% CI corresponds to the lower bound of the 95% 1-sided CI (the hypothesis is one-sided).

##### Subgroup PE analysis by age group

The above analysis of the first-time and overall malaria infections in the examination of the PE of SR will be based on all the subjects aged: ≥6 months to < 10 years. The same set of analysis will also be performed by two age subgroups: ≥6 months to 59 months old and 6 to < 10 years old to examine if the PE of SR will differ by two age groups.

##### PE analysis without baseline covariates

A PE analysis on the first-time and the overall infections will be also performed by removing all the baseline covariates from the cloglog models and only keeping “intervention group” as the only covariate (in addition to visit, as a categorical predictor per the model assumptions and set-up). The hazard ratios between SR and placebo will be provided, along with 2-sided 90% CIs.

##### Effects of SR on entomological endpoints

The endpoints in the entomological analysis will include the HBR (number of anopheline caught during the 12-hr interval overnight), anopheline parity rate, anopheline sporozoite rate, anopheline EIR, and the anopheline indoor density collected by light-trap.

We will report the frequencies and proportions of each mosquito Genus and species (anopheline and non-anophelines) collected using HLC and light trap methods across clusters and treatment arm.

The time profile plots of each aggregated entomological endpoint will be obtained over the baseline and intervention period. An appropriate statistical model for the HBR will be identified after examining the distributional characteristics of the HBR data, which are likely to follow (zero-inflated) Poisson distribution, or (zero-inflated) negative binomial distribution if there is over-dispersion. The covariates in the models will include the fixed effects of intervention, time, cluster population size, number of houses in a cluster, location (inside or outside), location and intervention interaction; and a random effect for cluster. The ratio between SR and placebo in HBR will be estimated. A similar model will be applied to analyze the density of mosquitoes caught by light traps. The ratio between SR and placebo on light trap density will be estimated.

The model for parity rate will be the (zero-inflated) Poisson distribution or a (zero-inflated) negative binomial distribution with the daily porous mosquitos as the outcome and the daily HBR as the offset, and the same set of covariates as those used in the model for analyzing HBR. The model for the sporozoite rate will be similar to the parity rate with the change of outcome variable to daily mosquitos with positive sporozoite. Note, if the data on parity and sporozoite positivity are highly unbalanced (not in terms of the distributions between the treatment arms, but the marginal distribution of the variable itself; e.g., 99% nulliparous or 99% negative sporozoite), then the model might lead to unstable estimates or the model might not even converge. In such cases, only summary statistics will be provided.

Summary statistics will be provided on EIR and the insecticide resistance at baseline and per year during the intervention period by treatment group.

##### Relationship between epidemiological and entomological endpoints for anopheline mosquitoes

To explore the relationship between the malaria hazard rate and the entomological endpoints, a similar model as the cloglog models used to address the primary objective on the first-time malaria infection and the secondary endpoint on the overall malaria infection will be applied to the epidemiological and entomological data in the clusters from which the entomological data are collected. The random effects and the individual-level and, HH-level covariates will the same as the cloglog models, and the cluster-level covariates will include the baseline incidence rate, cluster population size, and a covariate that captures the entomological information. Specifically, for HBR, the measurement to be paired with a malaria diagnosis in an individual is average daily HBR taken within 7 to 28 days before the diagnosis over the 2-week period and over the sentinel HHs where entomological endpoints are collected in the same cluster to which the individual belongs. The regression coefficient associated with log (HBR) quantifies the change in the malaria hazard rate on the log scale, given a one unit increase in log (HBR). For parity rate and sporozoite positivity rate, as long as there is enough data collected on these two endpoints and they are not highly unbalanced with regard to its marginal distribution (e.g., 99% mosquitos caught are nulliparous or sporozoite negative), the relationship between the malaria hazard rate and those two will also be investigated in a similar fashion as for HBR.

### Interim analyses {21b}

No interim analysis will be performed on the malaria and entomological data collected from the intervention period post randomization. The baseline data will be analyzed at the mid-point of the baseline period and at the completion of the baseline period to estimate the first-time and overall infection rates and their variability, and the mosquito density across the enrolled clustered.

### Methods for additional analyses (e.g. subgroup analyses) {20b}

#### Temporality of PE effects

It is expected that malaria incidence changes by seasonality (rainy vs dry) and year. To examine the temporality of malaria incidence rates and the PE effect, a supplementary analysis will be performed by adding the seasonality (Jun–Dec/wet/peak and Jan–May/dry/low) and year (1 and 2) and their interaction with intervention to the covariate list in the cloglog models used for analyzing the first-time and overall infections. The PE will be estimated by seasonality and year.

#### Human behavior adjusted PE analysis

The primary and secondary analyses for the first-time infection, the overall infection, and the examination of the relationship between the ento- and epi- endpoints will also be carried out by adjusting for the human behavior covariates the cloglog models, including “bednet usage” in the last 24 h (Y or N), “travel outside” (Y or N; an individual-level covariate), and the product application rate in each HH (expected to be close to 100%) if the data are balanced between the Y and N categories on “bednet usage” and “travel outside,” and there is practically/clinically meaningful variation in the product application rate across HHs and clusters.

#### Adjusted HBR analysis

The adjusted HBR at a given time point is calculated as the raw HBR× the proportion of people at the risk of being bitten in each HH. Specifically, in each HH where the HBR data are collected in the hourly interval from 6 pm to 6 am, the number of people indoors, the number of people outdoor, the number of people under bednet indoor, the number of people sleeping outdoor is also collected. The adjusted HBR indoor = raw HBR × number of subjects not under the protection of bednet/ total number of indoor subjects, and the adjusted HBR = raw HBR × number of subjects who sleep/total number of subjects outdoor. The analysis specified for estimating the effects of SR on the raw HBR will be applied to the adjusted HBR.

#### Per-protocol analysis

If the PP sample set differs from the ITT sample set, the primary analysis on the first-time infection and the secondary analysis on the overall infections will also be performed in the PP sample set.

### Methods in analysis to handle protocol non-adherence and any statistical methods to handle missing data {20c}

Standard Operating Procedures have been developed for all study activities and a Clinical Monitoring Plan has been established among the Sponsor, trial implementing partner, and the project Clinical Research Organization - fhiClinical. FhiClinical will be responsible for managing oversight on protocol adherence through interim monitoring visits during the study period. Departures from the protocol that is not participant-specific will be documented on a Protocol Deviation log developed by MRTC and reported as required, and the site re-educated as necessary. Any participant-specific non-compliances and other protocol deviations will be captured in the protocol deviation Case Report Form developed by MRTC and filed as hard copies. Major protocol deviations will be submitted via email to applicable IRBs and the Sponsor within 24 h of PI becoming aware of them, and followed by a detailed report, within 7 working days.

Significant effort will be made to avoid missing values on the outcome (malaria infection status and visit dates, entomological endpoints). When missing values occur for an outcome for reasons not related to the outcome, reasons for missingness and the missing fraction by treatment arm and cluster will be reported. Per protocol, the subjects are screened actively on their malaria status (the outcome) every 4 weeks.

If a subject misses one or more scheduled visits due to reasons not related to the SR product or the outcome, the subject will have missing values on the outcome that can be regarded as ignorable missingness (MAR or MCAR). If a subject drops out of study due to reasons unrelated to the SR product and/or malaria infection, then the missing observations from the subject can be regarded as ignorable missingness (MAR or MCAR). In both cases, all available data from the subject will be included in the primary and secondary analysis, without employing any specific technique to deal with the data.

Missing baseline covariates (individual-level, HH-level, cluster-level) that are a part of the regression models for the outcome of interest will be imputed using simple hot-deck imputation methods if the missing fraction for the covariate is < 5%> If the missing fraction for a covariable is ≥5%, appropriate multiple imputation approaches will be applied. If a non-ignorable portion of the subjects will have missing values on a covariate (due to missing at random or missing completely at random), that covariate may be excluded in the model.

### Plans to give access to the full protocol, participant level-data and statistical code {31c}

The statistical analysis plan (SAP) and analytic code will be made open access. Data and supporting information will be made available 12 months following completion of data analysis and will remain open access in the public domain.

## Oversight and monitoring

### Composition of the coordinating center and trial steering committee {5d}

UND will serve as the lead organization for this program and will assume the overall responsibility for management, oversight, and administration for the program. The coordinating personnel at UND will include the Lead PI, Scientific Director, Program Manager, Program Coordinator, and Finance Manager. UND will communicate on a day-to-day basis with CRS and MRTC. MRTC and CRS will be responsible for running the cRCT on a day-to-day basis which includes but will not be limited to conducting a baseline survey, deploying SRs, entomological monitoring, and subject follow-up. Representatives from MRTC, CRS, and UND will all serve on the data management team to oversee the development and implementation of data collection, recording, and cleaning.

fhiClinical will provide clinical oversight and monitoring of study processes, which includes but is not limited to checking enrolment, GCP training for study staff, ensuring subjects are properly consented, data are appropriately gathered, data quality, safety events are documented and reported as required, investigational product is managed and distributed per specifications, and study close-out activities occur on a timely basis.

### Composition of the data monitoring committee, its role and reporting structure {21a}

The DSMB reviews safety data about the Mali cRCT on an ongoing basis in order to monitor and rapidly identify any accumulating safety issues from across the program and may provide recommendations about stopping the study for safety reasons. Additionally, the DSMB provides additional credibility to study quality, by reviewing summary reports from PIs during baseline and intervention phases and making recommendations as needed about adjustments for study quality reasons. The DSMB consists of a Chair, Medical Monitor, DSMB statistician, and independent statistician.

Safety data should be reviewed routinely and regularly by the DSMB Medical Monitor. If significant concerns would be raised, he/she can engage with the committee. Summary of AEs, SAEs, and death reports observed during the studies should be reviewed by the entire committee at pre-determined checks (quarterly). This should include a comparison of the rate of AE and SAE in the two study arms and at the individual study site. The DSMB will be notified of any SAEs that are “at least possibly related” to the research study as they are reported to PIs. Unblinded efficacy data will be analyzed according to a pre-defined SAP by an Independent Statistician when pre-defined enrolment targets have been achieved. The role of the DSMB statistician will be to agree to what information will be reviewed (see below) and then review and interpret this information with the other DSMB members, perhaps to request further analysis, for example. The DSMB statistician will contribute input to program PIs as to what subset of the SAP is to be presented at different meetings.

Members generally have no ongoing financial relationship with a trial’s commercial sponsor and will not be involved in the trial conduct in any role other than that of a DSMB member. Prospective members will be asked to disclose their financial relationships with any of the sponsors and/or their competitors. The DSMB reports to the Sponsor, UND. The DSMB charter can be made available upon request to UND.

### Adverse event reporting and harms {22}

The SR product contains transfluthrin, a chemical used in currently available HH mosquito control products such as mosquito coils. Exposure to the product may cause mild eye and skin irritation, however, these effects are typically transient and disappear after time. The product may be harmful if chewed on or swallowed, so HH owners will be advised to keep it away from children. The SR product will be fixed at a position out of the reach of children and will be monitored at replacement to ensure it has not been moved. If a product is found to have been removed from its position in the HH, study staff will discuss with the HH owner to determine why it was removed and if there was any problem that led to its removal. Study staff will also reiterate safety precautions that should be taken in regard to the SR product.

AEs and SAEs of interest will be collected through passive surveillance by CHVs on cohort participants and other HH members receiving the study product. Additionally, AE and SAEs of interest will be solicited from study participants during follow-up visits (every 2 weeks) either in the house or clinic. We will also rely on passive surveillance of the outpatient registers at the local health facilities for reports of AE/SAE. Additionally, we may collect unsolicited reports during compound visits and/or product replacement. Anyone experiencing the SAEs or AEs of interest will be encouraged to seek care at the study clinic where clinical staff will attend to them at no cost.

An AE includes any noxious, pathological, or unintended change in anatomical, physiological, or metabolic functions as indicated by physical signs, symptoms, and/or laboratory detected changes occurring in any phase of the clinical study whether associated with the study intervention or placebo. This definition includes an exacerbation of pre-existing conditions or events, intercurrent illnesses. A SAE is any untoward medical occurrence that results in death, is life-threatening, results in persistent or significant disability/incapacity, requires in-patient hospitalization or prolongation of existing hospitalization, or is a congenital anomaly/birth defect in the offspring of a study subject. In addition, important medical events that may jeopardize the participant or may require intervention to prevent one of the other outcomes listed above will be considered serious.

All SAEs will be recorded on the appropriate SAE case report form (CRF), followed through resolution by a study physician, and reviewed by a study physician.

Symptomatic uncomplicated or severe malaria infection will be coded as AEs as will any other acute illness, throughout the 24 months of the study. Clinical malaria will be recorded in the same fashion as other AEs. In general, uncomplicated malaria will constitute an AE and severe malaria will constitute an SAE. Anticipated day-to-day fluctuations of pre-existing conditions that do not represent a clinically significant exacerbation need not be considered AEs. Discrete episodes of chronic conditions occurring during a study period will be reported as AEs to assess changes in frequency or severity. Pre-existing conditions or signs and/or symptoms (including any that are not recognized at study entry but are recognized during the study period) present in a participant prior to the start of the study will be recorded on the participant’s CRF.

AEs will be documented in terms of a medical diagnosis. When it is not possible to make a specific medical diagnosis, the AE will be documented in terms of signs and/or symptoms observed by the investigator or reported by the subject at each study visit. Any hospitalization will be considered a SAE.

AEs to be recorded as endpoints will be pre-defined based on SCJ. toxicology reports of “probable,” “possible,” “plausible,” and “unlikely.” “Probable”includes sensory irritation (oral and dermal). “Possible”includes nausea/vomiting (oral), skin irritation/rash (dermal), and runny nose (inhalation). “Plausible” includes salivation, and “unlikely” includes eye irritation and headache.

AEs and SAEs will be recorded throughout the study. SAEs and the study reports will also be provided to Mali’s Faculté de Médecine et d’Odonto Stomatologie - Faculté de Pharmacie (FMOS-FAPH) ethical committee as required by their current Standard Operating Procedures. Study teams will record AEs for every 2-week surveillance period at the time of house visit (during active malaria detection) and for the 30-day surveillance period at the time of clinic visits (during passive malaria detection). SAEs will be recorded at the time of death, hospitalization. Assessment of SAE relatedness to intervention will be conducted by the study clinician in-country with confirmation/verification by the DSMB Medical Monitor based on the study clinician SAE report and trends in other SAE datasets.

AEs and SAEs will be reported in future publications. Harms will be coded in accordance with MedDRA at time of safety outcome reporting. Summaries of symptom-based AEs, SAEs, and death reports observed during the studies will be reviewed by the trial DSMB at predetermined checks (quarterly). The AE/SAE will be labeled “Probable,” “Possible,” “Plausible,” or “Unlikely” due to SR. Summary statistics of AEs/SAEs, including mean, minimum, and maximum frequencies and percentages across clusters among enrolled subjects, will be provided by the treatment arm. Statistical comparisons of the AE/SAE rates between the two arms will be conducted upon the completion of the study. Two sets of statistical analyses will be run. One set will compare the proportion of having at least one occurrence in each symptom-based AE/SAE during the whole study between the two arms, and the other will compare the total number of occurrences for each AE/SAE between the two study arms. If the data collected permits meaningful statistical hypothesis testing, p-values from the treatment comparisons will be reported, with multiplicity correction via the FDR approach [21].

### Frequency and plans for auditing trial conduct {23}

FhiClinical will be responsible for clinical monitoring at the protocol implementation level, ensuring subjects will be properly consented, data will be appropriately gathered, safety events will be documented and reported as required, the investigational product will be managed and distributed per specifications, and study close-out activities will occur on a timely basis.

### Plans for communicating important protocol amendments to relevant parties (e.g., trial participants, ethical committees) {25}

Protocol amendments will be submitted to the Sponsor and local IRBs as well as the WHO Ethical Review Committee (ERC) for approvals. Any amendments, outcomes, analyses, or more will be communicated in person and virtually. Face-to-face meetings will be held between Lead PI, scientific director, site staff, and members of the site-specific countries where the trials are being conducted. These meetings will include National Malaria Control Programs, MOHs, other in-country public health officials, members of civil society, religious leaders, and key beneficiaries. These meetings will be critical as the trials will be in-progress and the topics addressed will be pertinent to facilitating further execution of the trial. Due to the project design and geographic location of the study team, teleconferences will be another method of formal project communication. Stakeholder teleconferences will be scheduled as needed to review and assess study progress and issues. Agendas for all teleconferences will be drafted by UND and distributed.

### Dissemination plans {31a}

Dissemination of results includes submission to WHO/VCAG, workshop with study partners, on-site meetings in Mali, and presentations at scientific meetings and/or peer-reviewed publications.

## Discussion

While the malaria burden has seen substantial reductions since 2000, malaria remains a serious public health concern, particularly in sub-Saharan Africa due to gaps in protection from ITNs and IRS. Supplemental tools such as SRs may address these gaps in coverage to further reduce the malaria burden. The role for SR products in malaria public health vector control are likely to be greatest in settings where early-evening and/or outdoor biting by *Anopheles* mosquitoes are not subject to the effects of ITNs and IRS or in complex emergencies where many people are housed in temporary shelters which are not conducive to ITN or IRS implementation. In addition, the active ingredient in SR products operates through a different mode of action and has demonstrated behavioral effects against both insecticide susceptible and resistant vectors responsible for transmitting multiple human pathogens [6]. Thus, SRs may serve as a tool in areas where insecticide resistance limits the effectiveness of ITNs and IRS and reducing selection pressure for insecticide resistance and thereby maintaining the tools’ natural life span [22].

There are thousands of registered SR products already in the market, adopted, and used for protection from nuisance biting; however, there is presently no public sector that uses SR products for disease control due to insufficient evidence for WHO policy recommendation. Over the past decade, national and international meetings have convened academics, industry, funders, and global public health experts, including WHO representatives, to discuss the role of SR products in the reduction of arthropod-borne diseases based on existing evidence.

A SR vector control product class is currently under WHO assessment for public health value, however, the biggest evidence gap is the lack of epidemiological data needed to demonstrate public health impact across a range of eco-epidemiological settings to inform a potential WHO policy recommendation for the incorporation of SR products into current multi-lateral disease control programs. In 2017, the WHO VCAG recommended additional clinical trials to evaluate SR against malaria in Africa [23]. These knowledge gaps must be addressed to inform WHO SR policy recommendations. This cRCT in Mali will serve as one of the additional trials to generate evidence to support WHO decision-making in regards to a recommendation.

If the WHO VCAG endorses a policy recommendation for the SR class to be recommended for public health use, national disease control programs will have the option to adopt a SR policy and “next-in-kind” SRs (e.g., with active ingredients to include other volatile pyrethroids such as metofluthrin) can be marketed within the public health channel without the need to undergo WHO VCAG assessment, incentivizing SR product research. Outputs will align with those of other global health stakeholders addressing residual malaria transmission, insecticide resistance, as well as access and barriers to market introduction of new vector control products. If the SR product is effective, it can be deployed to complement other vector control interventions such as ITNs to help mitigate the problem of insecticide resistance and times of vector biting where ITNs may be ineffective to further drive malaria towards elimination.

## Trial status

Under protocol version 8.2 from January 24, 2021, recruitment for the baseline cohort began July 8, 2021 and final subject enrolment for the baseline cohort has been completed on July 26, 2021.

The study has currently completed the baseline phase (as of January 15, 2022), whereby participants have been recruited, screened, enrolled, and followed up without intervention for 6 months. Baseline data analyses are ongoing to verify underlying assumptions of malaria incidence, coefficient of variation, and loss to follow-up. Recruitment, screening, and enrolment of subjects for follow-up with intervention are scheduled to be completed in March 2022.

## Supplementary Information

**Additional file 1.**

**Additional file 2.**

## Abbreviations

AE: Adverse events
AEGIS: Advancing Evidence for the Global Implementation of Spatial Repellents
AI: Active ingredient
AL: Artemether-lumefantrine
CI: Confidence interval
CRC: Center for Research Computing
cRCT: Cluster-randomized control trial
CRS: Catholic Relief Services
CV: Coefficient of variance
DSMB: Data Safety and Monitoring Board
EIR: Entomological inoculation rate
ERC: Ethical Review Committee
HBR: Human biting rate
HH: Household
HIN: Household identification number
HLC: Human landing catch
ICF: Informed consent form
IRS: Indoor residual spraying
ITN: Insecticide treated net
ITT: Intention to treat
LTFU: Loss to follow-up
MOH: Ministry of Health
MRTC: Malaria Research and Training Center
PE: Protective efficacy
PI: Principal Investigator
RDT: Rapid diagnostic test
SAE: Serious adverse event
SCJ: SC Johnson, A Family Company
SR: Spatial repellent
UND: University of Notre Dame
VCAG: Vector Control Advisory Group
WHO: World Health Organization
