# Supplementary material for: Evaluation of the protective efficacy of a spatial repellent to reduce malaria incidence in children in Mali compared to placebo: study protocol for a cluster-randomized double-blinded control trial (the AEGIS program)
Source: Trials. 2022 Apr 5;23:259. doi: 10.1186/s13063-022-06197-w (PMC8980511; doi:10.1186/s13063-022-06197-w)
Supplement: Supplementary file 1 — Additional file 1. [file 13063_2022_6197_MOESM1_ESM.pdf]

WHO Trial Registration Data Set (Version 1.3.1)

The minimum amount of trial information that must appear in a register in order for a given trial to be considered fully registered. There are currently 24 items in the WHO Trial Registration Data Set. It is sometimes referred to as the TRDS.

1. **Primary Registry and Trial Identifying Number**
   Name of Primary Registry, and the unique ID number assigned by the Primary Registry to this trial.

Primary registry: Clinicaltrials.gov
Trial record: NCT04795648
Available from:
https://clinicaltrials.gov/ct2/show/NCT04795648?term=grieco&draw=2&rank=6

2. **Date of Registration in Primary Registry**
   Date when trial was officially registered in the Primary Registry.

March 12, 2021

3. **Secondary Identifying Numbers**
   Other identifiers besides the Trial Identifying Number allocated by the Primary Registry, if any. These include:
   - The Universal Trial Number (UTN)
   - Identifiers assigned by the sponsor (record Sponsor name and Sponsor-issued trial number (e.g. protocol number))

University of Notre Dame's (Sponsor) Protocol number: 20-10-6245

   - Other trial registration numbers issued by other Registries (both Primary and Partner Registries in the WHO Registry Network, and other registries)
   - Identifiers issued by funding bodies, collaborative research groups, regulatory authorities, ethics committees / institutional review boards, etc.

   All secondary identifiers will have 2 elements: an identifier for the issuing authority (e.g. NCT, ISRCTN, ACTRN) plus a number.

   There is no limit to the number of secondary identifiers that can be provided.

Board Name: Ethical Committee of the Faculty of Medicine, Odonto-Stomatology and Pharmacy
Board Affiliation: Malaria Research and Training Center (MRTC), Faculty of Medicine, Dentistry and Pharmacy at the University, of Sciences, Techniques and Technologies of Bamako (USTTB), Mali
Approval Number: 2020/252/CE/FMOS/FAPH
Phone: (223) 20 22 52 77
Address:

Departement d'Epidemiologie des Affections Parasitaires (DEAP)
BP 1805 Bamako

WHO Ethical review Committee (WHO ERC) protocol ID: UND - Mali RCT

4. **Source(s) of Monetary or Material Support**
   Major source(s) of monetary or material support for the trial (e.g. funding agency, foundation, company, institution).

Unitaid

5. **Primary Sponsor**
   The individual, organization, group or other legal entity which takes responsibility for initiating, managing and/or financing a study. The Primary Sponsor is responsible for ensuring that the trial is properly registered. The Primary Sponsor may or may not be the main funder.

University of Notre Dame

6. **Secondary Sponsor(s)**
   Additional individuals, organizations or other legal persons, if any, that have agreed with the primary sponsor to take on responsibilities of sponsorship.

   A secondary sponsor may have agreed to:
   - take on all the responsibilities of sponsorship jointly with the primary sponsor; or
   - form a group with the Primary Sponsor in which the responsibilities of sponsorship are allocated among the members of the group; or
   - act as the Primary Sponsor's legal representative in relation to some or all of the trial sites.

N/A

7. **Contact for Public Queries**
   Email address, telephone number and postal address of the contact who will respond to general queries, including information about current recruitment status.

   "Note: The information provided in here is functional and not personal, it is recommended to provide institutional and not personal information. By providing this information the registrant consents that the information provided can or may be published on a public website. Once provided the information cannot be redacted or anonymized as

a result of new privacy legislation such as the European General Data Protection Regulation (GDPR)".

Notre Dame, IN 46556 USA
aegis@nd.edu

8. **Contact for Scientific Queries**
There must be clearly assigned responsibility for scientific leadership to a named Principal Investigator. The PI may delegate responsibility for dealing with scientific enquiries to a scientific contact for the trial. This scientific contact will be listed in addition to the PI.

"Note: The information provided in here is functional and not personal, it is recommended to provide institutional and not personal information. By providing this information the registrant consents that the information provided can or may be published on a public website. Once provided the information cannot be redacted or anonymized as a result of new privacy legislation such as the European General Data Protection Regulation (GDPR)".

The contact for scientific queries must include:
- Name and title, email address, telephone number, postal address and affiliation of the Principal Investigator, and;
- Email address, telephone number, postal address and affiliation of the contact for scientific queries about the trial (if applicable). The details for the scientific contact may be generic (that is, there does not need to be a named individual): e.g. a generic email address for research team members qualified to answer scientific queries.

Principal Investigator and for scientific queries:

Dr. John P. Grieco
Eck Institute for Global Health
University of Notre Dame
243 Galvin Life Science
Notre Dame, IN 46556
jgrieco@nd.edu
+1 (574) 631-7572

9. **Public Title**
Title intended for the lay public in easily understood language.

Spatial Repellents for Vector Control (AEGIS Mali RCT).

10. **Scientific Title**
    Scientific title of the study as it appears in the protocol submitted for funding and ethical review. Include trial acronym if available.

Spatial Repellent Products for Control of Vector Borne Diseases.

11. **Countries of Recruitment**
    The countries from which participants will be, are intended to be, or have been recruited at the time of registration.

Mali

12. **Health Condition(s) or Problem(s) Studied**
    Primary health condition(s) or problem(s) studied (e.g., depression, breast cancer, medication error).

    If the study is conducted in healthy human volunteers belonging to the target population of the intervention (e.g. preventive or screening interventions), enter the particular health condition(s) or problem(s) being prevented.

Malaria

13. **Intervention(s)**
    For each arm of the trial record a brief intervention name plus an intervention description.

    Intervention Name: For drugs use generic name; for other types of interventions provide a brief descriptive name.
    - For investigational new drugs that do not yet have a generic name, a chemical name, company code or serial number may be used on a temporary basis. As soon as the generic name has been established, update the associated registered records accordingly.
    - For non-drug intervention types, provide an intervention name with sufficient detail so that it can be distinguished from other similar interventions.

    Intervention Description: Must be sufficiently detailed for it to be possible to distinguish between the arms of a study (e.g. comparison of different dosages of drug) and/or among similar interventions (e.g. comparison of multiple implantable cardiac defibrillators). For example, interventions involving drugs may include dosage form, dosage, frequency and duration.

    If the intervention is one or more drugs then use the International Non-Proprietary Name for each drug if possible (not brand/trade names). For an unregistered drug, the generic name, chemical name, or company serial number is acceptable.

    If the intervention consists of several separate treatments, list them all in one line separated by commas (e.g. "low-fat diet, exercise").

For controlled trials, the identity of the control arm should be clear. The control intervention(s) is/are the interventions against which the study intervention is evaluated (e.g. placebo, no treatment, active control). If an active control is used, be sure to enter in the name(s) of that intervention, or enter "placebo" or "no treatment" as applicable. For each intervention, describe other intervention details as applicable (dose, duration, mode of administration, etc).

The intervention arm will have a new formulation of transfluthrin, Mosquito Shield™, a passive emanator that releases active ingredient over a period of up to four weeks.
A placebo product of matched design with inert ingredients will be applied similarly in the control arm.

14. **Key Inclusion and Exclusion Criteria**

Inclusion and exclusion criteria for participant selection, including age and sex. Other selection criteria may relate to clinical diagnosis and co-morbid conditions; exclusion criteria are often used to ensure patient safety.

If the study is conducted in healthy human volunteers not belonging to the target population (e.g. a preliminary safety study), enter "healthy human volunteer".

Inclusion Criteria:

- Children ≥ 6 months to < 10 years of age
- Children with Hb > 7 mg/dL and no other serious illness
- Sleeps in cluster ≥ 90% of nights during any given month
- Not participating in another clinical trial investigating a vaccine, drug, medical device, or a medical procedure during the trial
- Provision of informed consent (and/or assent) form (ICF) signed by the parent(s) or guardian

Exclusion Criteria:

- Children < 6 months or ≥ 10 years
- Children with Hb ≤ 7 mg/dL, or Hb < 6mg/dL with signs of clinical decompensation and no other serious illness
- Sleeps in cluster <90% of nights during any given month
- Participating or planned participation in another clinical trial investigating a vaccine, drug, medical device, or a medical procedure during the trial
- No provision of ICF (and/or assent) signed by the parent(s) or guardian

15. **Study Type**
    Study type consists of:
    - Type of study (interventional or observational)

Interventional (Clinical Trial)

- Study design including:
    - Method of allocation (randomized/non-randomized)

Randomized

- Masking (is masking used and, if so, who is masked)

Quadruple (Participant, Care Provider, Investigator, Outcomes Assessor)

- Assignment (single arm, parallel, crossover or factorial)

Parallel Assignment. The study design will be a prospective cluster Randomized Control Trial (cRCT).

- Purpose

Prevention

- Phase (if applicable)

For randomized trials: the allocation concealment mechanism and sequence generation will be documented.

16. **Date of First Enrollment**
    Anticipated or actual date of enrolment of the first participant.

July 8, 2021

17. **Sample Size**
    Sample Size consists of:
    - Number of participants that the trial plans to enrol in total.

1,920

- Number of participants that the trial has enrolled.

1,911 participants enrolled as of December 20, 2021.

18. **Recruitment Status**
    Recruitment status of this trial:
    - Pending: participants are not yet being recruited or enrolled at any site
    - Recruiting: participants are currently being recruited and enrolled
    - Suspended: there is a temporary halt in recruitment and enrolment
    - Complete: participants are no longer being recruited or enrolled
    - Other

Recruiting

19. **Primary Outcome(s)**
    Outcomes are events, variables, or experiences that are measured because it is believed that they may be influenced by the intervention.

    The Primary Outcome should be the outcome used in sample size calculations, or the main outcome(s) used to determine the effects of the intervention(s). Most trials should have only one primary outcome.

    For each primary outcome provide:
    - The name of the outcome (do not use abbreviations)
    - The metric or method of measurement used (be as specific as possible)
    - The timepoint(s) of primary interest

    Example:
    Outcome Name: Depression
    Metric/method of measurement: Beck Depression Score
    Timepoint: 18 weeks following end of treatment

Primary Outcome Measure:

1. Number of first-time malaria infection (Plasmodium falciparum (Pf)) during intervention period. [Time Frame: 24 months].

   Measured by microscopy in children aged between 6 months to 10 years.

20. **Key Secondary Outcomes**
    Secondary outcomes are outcomes which are of secondary interest or that are measured at timepoints of secondary interest. A secondary outcome may involve the same event, variable, or experience as the primary outcome, but measured at timepoints other than those of primary interest.

    As for primary outcomes, for each secondary outcome provide:
    - The name of the outcome (do not use abbreviations)
    - The metric or method of measurement used (be as specific as possible)
    - The timepoint(s) of interest

Secondary Outcome Measures:

1. Number of overall new Plasmodium falciparum malaria infections during intervention period. [Time Frame: 24 months]
   Measured by microscopy in children aged between 6 months to 10 years.

2. Number of first-time Plasmodium falciparum malaria infections by two age groups (≤ 59 months old; 5 years old to 10 years old). [Time Frame: 24 months]
   Measured by microscopy in children aged between 6 months to 10 years.

3. Number of overall Plasmodium falciparum malaria infections by two age groups (≤ 59 months old; 5 years to 10 years old). [Time Frame: 24 months]
   Measured by microscopy in children aged between 6 months to 10 years.

4. Anopheline-human contact (indoor and outdoor) using human biting rate (HBR) as an indicator for all anophelines and by anopheline species. [Time Frame: 24 months]
   Measured by human-landing catch (HLC) during 12-h intervals on a quarterly basis during intervention period.

5. Anopheline parity rate as an indicator of population age structure for all anophelines and by anopheline species. [Time Frame: 24 months]
   Measured by mosquito ovarian dissections from a sub-sample of anophelines collected during HLC procedures during intervention period.

6. Anopheline infectivity using sporozoite rate as an indicator for all anophelines and by anopheline species. [Time Frame: 24 months]
   Measured by laboratory detection of sporozoites in mosquito head-preps from a sub-sample of anophelines collected during HLC and/or CDC-light trap procedures during intervention period.

7. Anopheline infectivity using entomological inoculation rate (EIR) as an indicator for all anophelines and by anopheline species. [Time Frame: 24 months]
   Measured by calculating the number of sporozoite-infected anopheline mosquitoes captured per person during intervention period from HLC and/or CDC-light trap procedures.

8. CDC-light trap indoor density for all anophelines and by anopheline species. [Time Frame: 24 months]
   Measured by CDC-light trap collections during 12-h intervals on a monthly basis during intervention period.

9. Insecticide resistance. [Time Frame: 30 months]
   Measured by WHO filter paper test and CDC bottle assays during baseline and intervention period.

10. Adverse Events and Serious Adverse Events. [Time Frame: 30 months]
    Measured by solicited and unsolicited reports during baseline and intervention period. Mean, minimum and maximum frequency and percentage of Adverse Events(AEs) and

Severe Adverse Events (SAEs) across clusters among enrolled subjects will be summarized by treatment arm.

21. **Ethics Review**
    The ethics review process information of the trial record in the primary register database. It consists of:
    - Status (possible values: Not approved, Approved, Not Available)
    - Date of approval
    - Name and contact details of Ethics committee(s)

Board Name: Ethical Committee of the Faculty of Medicine, Odonto-Stomatology and Pharmacy
Board Affiliation: Malaria Research and Training Center (MRTC), Faculty of Medicine, Dentistry and Pharmacy at the University, of Sciences, Techniques and Technologies of Bamako (USTTB), Mali
Approval Number: 2020/252/CE/FMOS/FAPH
Phone: (223) 20 22 52 77
Address:
Departement d'Epidemiologie des Affections Parasitaires (DEAP)
BP 1805 Bamako

First Approval November 12, 2020 of the protocol version 8.1 from October 29, 2020 and latest amendment (2nd) approved July 1, 2021 for the protocol version 8.2 from January 24, 2021 and ICF v8.3 from June 29, 2021.

22. **Completion date**
    Date of study completion: The date on which the final data for a clinical study were collected (commonly referred to as, "last subject, last visit").

December 2023 anticipated.

23. **Summary Results**
    It consists of:
    - Date of posting of results summaries
    - Date of the first journal publication of results
    - URL hyperlink(s) related to results and publications
    - Baseline Characteristics: Data collected at the beginning of a clinical study for all participants and for each arm or comparison group. These data include demographics, such as age and sex, and study-specific measures.

- Participant flow: Information to document the progress and numbers of research participants through each stage of a study in a flow diagram or tabular format.
- Adverse events: An unfavorable change in the health of a participant, including abnormal laboratory findings, and all serious adverse events and deaths that happen during a clinical study or within a certain time period after the study has ended. This change may or may not be caused by the intervention being studied.
- Outcome measures: A table of data for each primary and secondary outcome measure and their respective measurement of precision (eg a 95% confidence interval) by arm (that is, initial assignment of participants to arms or groups) or comparison group (that is, analysis groups), including the result(s) of scientifically appropriate statistical analyses that were performed on the outcome measure data, if any.
- URL link to protocol file(s) with version and date
- Brief summary

N/A

24. **IPD sharing statement**
    Statement regarding the intended sharing of deidentified individual clinical trial participant-level data (IPD). Should indicate whether or not IPD will be shared, what IPD will be shared, when, by what mechanism, with whom and for what types of analyses. It consists of:
    - Plan to share IPD (Yes, No)
    - Plan description

The analytical data will be anonymized and GPS tag-blurred to remove sensitive information prior to sharing. The data and supporting information will be made available 12 months following completion of data analysis and will remain open access in the public domain.
